# *Bartonella henselae* in African Lion, South Africa

**DOI:** 10.3201/eid1012.031054

**Published:** 2004-12

**Authors:** Anne-Marié Pretorius, Johannes M. Kuyl, Diana R. Isherwood, Richard J. Birtles

**Affiliations:** * University of the Free State, Bloemfontein, South Africa;; † University of Liverpool, Liverpool, United Kingdom;; ‡ University of Liverpool, Neston, United Kingdom

**Keywords:** letter, Panthera leo, Bartonella henselae, isolation, South Africa

**To the Editor:** Four members of the bacterial genus *Bartonella*, *Bartonella henselae*, *B. clarridgeiae*, *B. koehlerae*, and *B. bovis*, have been isolated from felids, mostly domestic cats (*1**,**2*). Of these four species, *B. henselae* and *B. clarridgeiae* are recognized human pathogens, which cause many illnesses, including endocarditis, prolonged fever, various ocular infections and, most commonly, cat scratch disease (*1*).

In 1994, domestic cats (*Felis domesticus*) were found to be a reservoir for *B. henselae*; subsequent surveys have shown that a large proportion of the domestic cat population worldwide has been exposed to, or infected with, bartonellae (*1*). The epidemiologic features of *Bartonella* infection in other felid species has been explored; a high prevalence of seropositivity has been found in free-ranging and captive wild cats from California and Florida (*3*), as well as panthers from Florida (*4*). *B. henselae* has been isolated from a captive cheetah in Zimbabwe (*5*).

During 2002, blood samples were collected from 65 African lions that inhabited three ranches in the Free State Province of South Africa. These ranches breed and rear lions specifically for game. Although the lions are contained within vast (several km^2^) enclosures, they are free to move about and interact with one another. The lions have minimal contact with humans or other animals, except carcasses of horses and donkeys that are provided as food. The lions do not receive any other food, food supplements, growth enhancers, or antiparasite prophylaxis. All three ranches are deep in the veld, at least 20 km from any settlements. Blood samples were drawn from the lions as part of an ongoing health surveillance program conducted by the African Large Predator Research Unit, University of Bloemfontein. Whole blood samples were drawn aseptically from each lion into EDTA tubes, stored at 4°C before being returned to the laboratory, and then frozen at –70°C in the laboratory. Subsequently, blood samples were thawed, and an aliquot was plated onto 10% sheep blood–enriched agar and incubated at 37°C in a 5% CO_2_ atmosphere for a maximum of 45 days. One culture yielded putative bartonellae (small, smooth, white-gray colonies) after 14 days' incubation. A crude DNA extract was prepared from this isolate and used as a template in previously described polymerase chain reaction–based assays to detect and identify *Bartonella* species which targeted fragments of the 16S rRNA encoding gene and 16S/23S intergenic spacer region (*6*). Amplification products of the expected size were obtained from the DNA extract. The nucleotide base sequence of each product showed that each shared 100% similarity with sequences of other *B. henselae* isolates held in GenBank. The 16S rRNA gene sequence was identical to that of type II variants.

Antisera from 62 of the 65 samples were tested for the presence of anti-*Bartonella* immunoglobulin G antibodies using an enzyme-linked immunosorbent assay previously evaluated to detect antibodies in domestic cats (*7*). Eighteen of the samples had matrix scores above the upper limit of the normal range of values observed in uninfected cats, thus indicating past exposure to *Bartonella* species. No serum from the *B. henselae* culture-positive animal was available for testing.

Our findings confirm that lions are susceptible to infection by *B. henselae*, but their role as reservoirs for this species remain unclear. The observed prevalence of infection (1.5%) and exposure rate (29%) in our study are lower than those typically observed in domestic cats, particularly in warmer regions of the world. Nonetheless, our serologic data do suggest that a substantial proportion of the lions are exposed to bartonellae. Although limited, our assessment of the lion *B. henselae* isolate suggests that it is within the genetic spectrum of strains associated with domestic cats, and lions may serve as an extension to this reservoir. The extent of contact between domestic cats, or their ectoparasites, and the farmed lions we studied is likely to be minimal, given the remoteness of the enclosures (the infected lion lived on a cat-free ranch). However, the lions may have contact with other wild-living felids such as the African wild cat (*Felis silvestris lybica*), small spotted cat (*Felis nigripes*), and the caracal (*Caracal caracal*) which are endemic to the region.
